# Reduced expression of the PER2 protein contributes to β _1_-AA-induced cardiac autophagy rhythm disorders: Role of PER2 in autophagy rhythm disorders

**DOI:** 10.3724/abbs.2025023

**Published:** 2025-04-10

**Authors:** Pengjia Li, Jiayan Feng, Jiao Guo, Jin Xue, Yang Li, Shiyuan Wen, Xiaohui Wang, Huirong Liu, Li Wang

**Affiliations:** 1 Research Institute of Circadian Rhythm and Disease Shanxi Medical University Taiyuan 030001 China; 2 Department of Pathology Yuncheng Central Hospital Yuncheng 044000 China; 3 School of Basic Medical Sciences Capital Medical University Beijing 100069 China; 4 Key Laboratory of Cellular Physiology Shanxi Province Taiyuan 030001 China

**Keywords:** β _1_-AA, autophagy rhythm, biological clock, PER2, mTORC1

## Abstract

Heart failure may be linked to fluctuations in the rhythm of autophagy in cardiomyocytes throughout the day. Circadian rhythms depend on the regulation of core biological clock proteins, with PER2 playing a crucial role. Our previous research confirmed that the presence of β _1_-adrenergic receptor autoantibodies (β _1_-AAs) could inhibit myocardial autophagy, leading to cell death and heart failure. However, it remains unclear whether β _1_-AA induces cardiac autophagy rhythm disorders by affecting PER2 expression. In this study, we find that β _1_-AA disrupts the autophagy rhythm in cardiomyocytes, which is primarily indicated by decreased expression of the autophagy marker protein LC3. β _1_-AA disrupts the rhythmic expression of the PER2 protein in myocardial cells, which is manifested mainly by a decrease in PER2 protein expression. Metoprolol is used to verify that the β _1_-adrenergic receptor contributes to the reduction in the Per2 protein caused by β _1_-AA. Knockdown of *Per2* with lentivirus reduces the inhibition of LC3 expression caused by β _1_-AA, whereas overexpression of Per2 in cardiomyocytes using lentivirus significantly restores the β _1_-AA-induced decrease in LC3 expression. Moreover, mTORC1 activation is found to participate in β _1_-AA-induced autophagy inhibition in cardiomyocytes after pretreatment with the mTORC1 inhibitor rapamycin. Furthermore, the decreased expression of the PER2 protein caused by β _1_-AA disrupts the myocardial autophagy rhythm by promoting mTORC1 activation through lentiviruses that knock down or overexpress the *Per2* gene. This study provides an experimental basis for the precise treatment of cardiovascular diseases from the perspective of biological rhythm.

## Introduction

Heart failure is the final stage of various cardiovascular diseases [1]. With the increasing aging of society, the prevalence and mortality of heart failure worldwide are increasing, with China accounting for 1/3 of all heart failure cases [ 2, 3]. Lifestyles related to circadian rhythm disturbances, such as “daily staying up late”, “shift work” and “sleep disorders”, can disrupt the repair cycle of the heart and cause damage to or even death of cardiomyocytes, resulting in an increased risk of heart failure or aggravation of pre-existing heart failure [4]. Previous studies have shown that various biological activities that maintain the normal operation of cells, including autophagy, undergo circadian changes [5]. Adverse cardiac events occur frequently in the early morning, when autophagy activity is low [6], suggesting that the occurrence of heart failure may be related to the circadian rhythm of autophagy in cardiomyocytes throughout the day. Therefore, it is particularly important to identify the factors that disturb the circadian rhythm of autophagy during heart failure.

Clinical data show that autoantibodies are present in the serum of 40%–60% of patients with heart failure and continuously activate the β _1_-adrenergic receptor (β _1_-AR), namely, β _1_-adrenergic receptor autoantibodies (β _1_-AAs) [7]. The removal of β _1_-AA from the serum of patients with heart failure by the immunosorbent technique could significantly improve the cardiac function of patients [8]. Our previous studies revealed that β _1_-AA can reduce the level of myocardial autophagy and promote cardiac cell death, leading to heart failure [9]. However, it is not clear whether β _1_-AA is a factor causing the disturbance of the rhythm of cardiomyocyte autophagy.

A previous study showed that the autophagy rhythm is regulated by the circadian rhythm [10]. The circadian rhythm is a regulated rhythmic oscillation with a 24-h cycle that exists universally in the body. Its molecular mechanism involves a transcriptional translation feedback loop composed of a series of clock genes [11], in which the negative feedback regulation of the PER2 protein plays a key role [12]. Per2 is closely related to autophagy [13]. After the *Per2* gene is silenced in fibroblasts, the levels of the autophagy-related proteins LC3II and ULK1 are significantly decreased, and their rhythms are also significantly abnormal [14]. Therefore, we speculated that β _1_-AA could disturb the autophagy rhythm of cardiomyocytes by altering the expression of PER2 in cardiomyocytes and further explored the specific mechanism from the perspective of mTORC1 to provide a new experimental basis for the precise treatment of cardiovascular diseases from the perspective of biological rhythm.

## Materials and Methods

### Animals and cells

Male C57BL/6 mice (aged 6–8 weeks, weighing 18–22 g) and male SD rats (aged 6–8 weeks, weighing 180–210 g) were purchased from the Laboratory Animal Center of Shanxi Medical University [Animal Qualification Certificate No. SCXK (Jin) 2015-0001]. H9c2 rat cardiomyocytes were purchased from the Shanghai Cell Bank, Chinese Academy of Sciences (SCSP-5211; Shanghai, China).

### Experimental reagents

β _1_-AR-ECII antigen peptide was purchased from China Gill Biochemical (Shanghai, China); DMEM (high sugar) was purchased from Gibco (C11995500BT; Carlsbad, USA); the primary antibodies were purchased from Abcam (Cambridge, UK); the secondary antibodies were purchased from Zhongshan Jinqiao Company (Beijing, China); and the dexamethasone was from Solarbio (D6950; Beijing, China).

### Establishment of an active immune animal model

Male C57BL/6 mice aged 6 to 8 weeks were randomly selected and divided into a control group and a β _1_-AR-active immune group according to the random number table method. In the active immunization (β _1_-AA) group, a β _1_-AR-ECII antigen mixed emulsion at a dose of 0.4 μg/g was injected into the subcutaneous back of each mouse, which was the first immunization, and the boost immunization was performed every 2 weeks for a total of 6 weeks. In the solvent control group, the same dose of Na _2_CO _3_ and adjuvant mixed solution was injected into the subcutaneous back of each mouse, and the immunization time and frequency were the same as those of the active immunization group.

### Rhythm experiment procedure and sample collection

The mice were randomly divided into a solvent control group and a β _1_-AR-ECII primary immunization group to detect endogenous circadian rhythm changes. After the first immunization, the mice were placed in a 12-h light/12-h dark (LD) cycle environment for synchronization for 2 weeks. After the booster immunization, the light source was turned off, and the LD environment was changed to continuous darkness (DD) for an additional 2 weeks. Circadian Time 0 (CT0) was referenced to the extrapolated lights-on time from the prior LD cycle, with myocardial sampling initiated at CT0. Sampling time points were designated at CT0, CT4, CT8, CT12, CT16, and CT20, with a 4-h interval between each point. Approximately 20 mg of myocardial tissue was collected at each time point for subsequent experimental analysis.

The sampling procedure for H9c2 cardiomyocytes is as follows: Cells were seeded until they reached 70%–80% confluency, and dexamethasone (Dex) was added at a final concentration of 0.1 μM to synchronize myocardial cells. After synchronization, samples were collected at 4-h intervals (CT0, CT4, CT8, CT12, CT16, and CT20). The collected cell samples were used for subsequent experimental analysis.

### Concentration and purification of β _1_-AA

The rats were injected via the same active immunization method, and the serum of the successfully immunized rats was mixed and concentrated in a special Eppendorf tube. The β _1_-AA in the serum was subsequently purified via an affinity chromatography column (17112801; Cytiva, Shanghai, China), and its concentration was detected for subsequent cell experiments.

### SA-ELISA

The content of β _1_-AA in the serum of actively immunized mice was detected by SA-ELISA, and the β _1_-AA in the serum of antibody-positive mice was purified to prove that the active immunized mouse model of β _1_-AR was successful. The synthetic β _1_-AR-ECII antigen peptide was prepared in a solution and coated uniformly on the bottom of the microplate to produce a solid-phase antigen. Isolation was carried out in sequence, followed by incubation of the serum to be tested, incubation with the biotin-labeled secondary antibody (ZB-2040 and ZB2020; Zhongshan Jinqiao Company), incubation with the horseradish peroxidase (HRP)-labeled streptavidin lecithin (ZB-2404; Zhongshan Jinqiao Company), and incubation with a chromogenic solution (Citric Acid, GB-9855-88; East China University of Chemical Technology Fangzhuang Third Auxiliary Factory, Shanghai, China; Disodium Hydrogen Phosphate, GB/T-1263-1986, Tianjin Fengchuan Chemical Reagent Co., Ltd., Tianjin, China; ABTS, A9590; Solarbio; H _2_O _2_, Cat No: AR1108; Boster Biological Technology, Wuhan, China). Finally, the absorbance value was detected with a microplate reader (Beijing Shengyuan Chengye Technology Co., Ltd.‌‌, Beijing, China).

### Cell culture

H9c2 rat cardiomyocytes were cultured in a 5% CO _2_ incubator (Eppendorf, Hamburg, Germany) at 37°C. When the cells were in good condition, they were treated as follows. Control group: after dexamethasone treatment for 4 h, H9c2 cells were treated with purified negative control IgG (1 μM). β _1_-AA group: after dexamethasone treatment for 4 h, purified β _1_-AA was added to H9c2 cells at a final concentration of 1 μM.

### Cell cycle analysis by flow cytometry

The cell cycle distribution was measured by flow cytometry to determine the degree of cell synchronization. H9c2 cardiomyocytes were uniformly spread in 6-well plates, and when the cell density reached 60%–70%, dexamethasone (final concentration of 100 nM) was added and incubated for 1, 2, and 4 h, and the cells were collected and placed in a centrifuge tube containing 75% ethanol for low-temperature fixation. Then, dye (Cell Cycle Staining kit, CCS01; MultiSciences (Lianke) Biotech Co., Ltd., Hangzhou, China) that can specifically bind to DNA was added to the cell suspension for flow cytometric detection. The different fluorescence intensities shown in the results indicated different proportions of cells at different stages. The proportion of G0/G1 phase cells is an indicator of whether the cells have reached synchronization.

### Western blot analysis

The protein expression levels in myocardial tissue and cells were detected by western blot analysis. First, proteins were extracted from the collected mouse tissues or cells using ultrasonic crusher (SONICS & MATERLALS Company, Newtown, USA), and the protein concentration was detected using BCA Protein Assay kit (AR0416; Boster Biological Technology). The samples (40 μg) were separated by polyacrylamide gel electrophoresis (SDS-PAGE; P1204-50T; Solarbio) and transferred onto a PVDF (IPVH00010; Millipore, Billerica, USA) membrane. The membrane was blocked in skim milk solution for 2 h. Then primary antibody was added and incubated at 4°C overnight, followed by incubation with the secondary antibody for 2 h. The protein bands on the membrane were visualized using supersensitive luminescent solution (MA0816-1; Dalian Meilun Biotechnology Co., Ltd., Dalian, China) and placed in the exposure instrument (Bio-Rad, Hercules, USA) for imaging. Finally, imaging software (Image J) was used to analyze the signal intensities.

### Immunofluorescence staining

Immunofluorescence staining was used to detect the expression and localization of PER2 protein in cells. After the cells successfully climbed the tablet, they were treated with drugs. The samples were fixed with paraformaldehyde, the samples were broken with Triton X-100 (T8200; Solarbio), the samples were sealed with 5% BSA (SW3015; Solarbio), and the corresponding primary and fluorescent secondary antibodies were added (avoiding light). Finally, after DAPI (G1012; Servicebio, Wuhan, China) nucleation, protein fluorescence was observed via a confocal laser microscope (Olympus, Tokyo, Japan).

### CCK-8 assay

One hundred microliters of cell suspension was inoculated into 96-well cell culture plates, and the number of cells per well was controlled at 20%–30%. The culture plate was placed at 37°C and incubated in a 5% CO _2_ incubator for 24 h. Then, the PBS was removed, and the medium was changed. A certain volume of the drug to be tested was added to the culture plate and incubated for 24 h. Finally, 10 μL of CCK-8 reagent (BMU106-CN; Dojindo Laboratories, Kyushu Island, Japan) was added to each well, and after incubation in the incubator for 2 h, the absorbance value at 450 nm was measured via a microplate reader (Beijing Shengyuan Chengye Technology Co., Ltd.). The calculation formula was as follows: relative cell viability (%) = (experimental group absorbance value – blank control group absorbance value)/(negative control group absorbance value–blank control group absorbance value) × 100%. The percentage obtained from each set of data is presented as the cell survival rate.

### Statistical analysis

The SPSS 16.0 statistical package was used analyze the data and generate a normal distribution curve. Data are presented as the mean ± standard deviation. The difference in protein expression between the control group and the experimental group was analyzed by paired sample *t* tests. Moreover, one-way analysis of variance was used to detect the independence of multiple sets of data. Values with *P*  < 0.05 were considered statistically significant. JTK_CYCLE is a new nonparametric statistical algorithm for detecting and describing the rhythm portion of large-scale genomic data. JTK_CYCLE analysis was performed via the Windows R 2.10.0 software package, the 95% confidence interval was set, and the JTK_ *P* value in each group of output data was recorded ( *P*  < 0.05 after correction indicates that it has circadian fluctuations).

## Results

### β _1_-AA disrupts the myocardial autophagy rhythm, mainly by reducing the expression of the autophagy marker protein LC3

**Figure 1:**
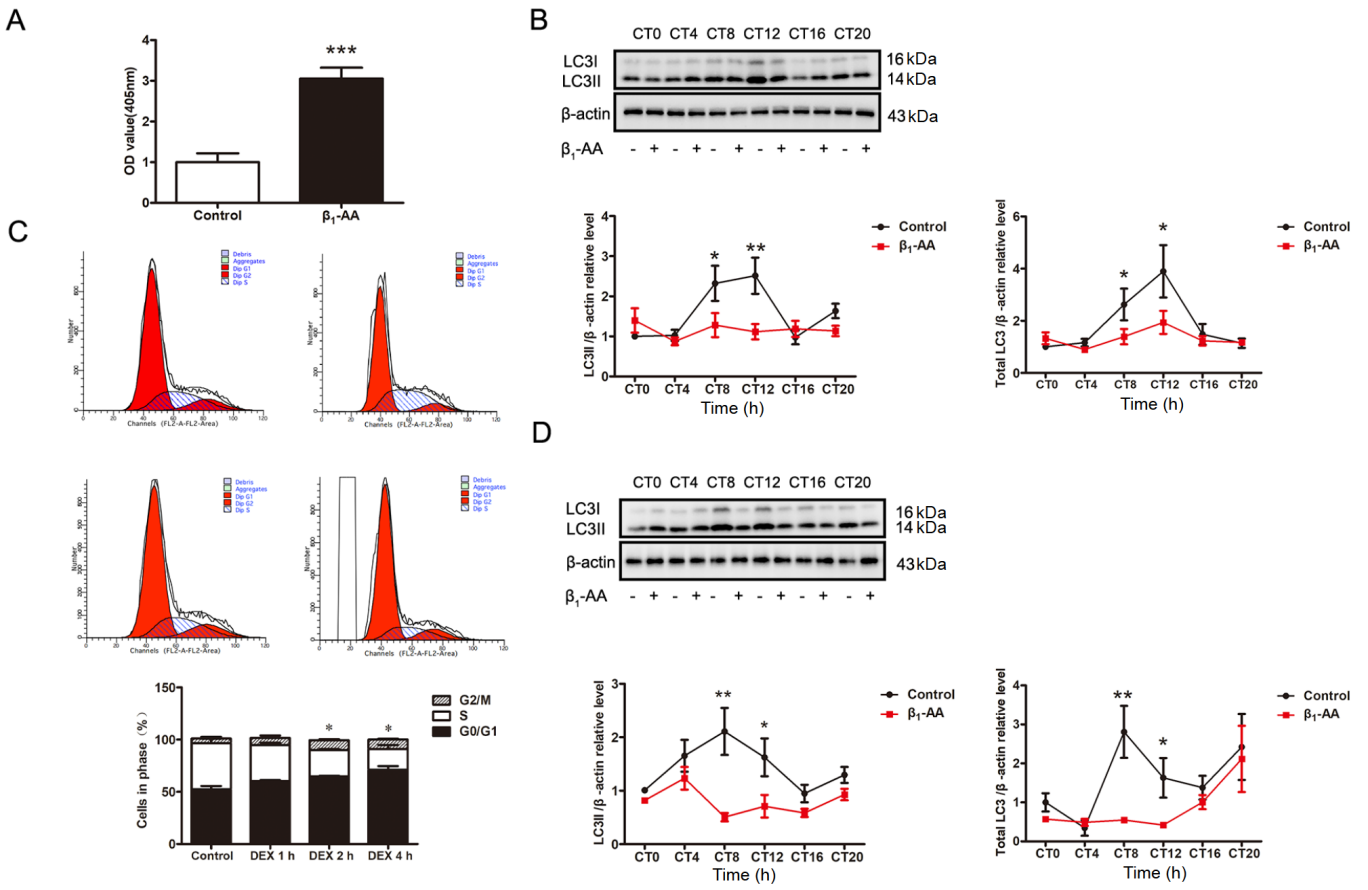
Figure 1 Rhythmic expression disturbance of autophagy marker protein LC3 induced by β1-AA (A) The serum β1-AA antibody content (OD value) of mice in the control group and active immune group was detected by SA-ELISA. (B) Western blot analysis of total LC3 and LC3II expressions in myocardial tissue of mice in the control group and β1-AA group at different time points (n = 14). (C) The cell distribution cycle of the control group was detected by flow cytometry after dexamethasone treatment for 1 h, 2 h and 4 h. (D) Western blot analysis of total LC3 and LC3II expressions in cardiomyocytes of the control group and β1-AA group at different time points (n = 7). *P < 0.05, **P < 0.01, ***P < 0.001 vs control group.

To detect the endogenous autophagy rhythm of myocardial tissue, the mice were actively immunized with β _1_-AR-ECII, and the immunization was boosted once every 14 days. After the first immunization, the mice in each group were placed in an L/D environment (light/dark for 12 h) for 2 weeks. After the second immunization, the environment was adjusted to continuous darkness (DD) for 2 weeks. CT0 was defined as the light onset. SA-ELISA revealed that the level of β _1_-AA in the serum of 4-week-old mice was significantly increased ( Figure 1A), indicating that the model was successfully constructed. Western blot analysis was performed to detect the expression of LC3 protein, a key autophagy marker, in the myocardial tissue of control group mice. The results revealed that LC3II protein expression (LC3II/β-actin) in the myocardium of the mice in the control group exhibited a significant circadian rhythm (JTK_CYCLE ADJ, *P*  < 0.05) ( Table 1). Compared with that in the control group, LC3II protein expression in the myocardial tissue of actively immunized mice was significantly lower at CT8 and CT12. In order to more comprehensively evaluate whether the decrease in LC3II protein expression induced by β _1_-AA active immunization was influenced by total LC3 protein expression, we further analyzed the total LC3 protein expression. Total LC3 protein expression was quantified as the sum of LC3I and LC3II normalized to β-actin, expressed as (LC3I + LC3II)/β-actin. The results showed that the expression of total LC3 were consistent with the trend of LC3II. This experiment further supported that β _1_-AA not only inhibited the conversion of LC3I to LC3II, but also down-regulated the expression of total LC3 protein ( Figure 1B). That is to say, β _1_-AA can lead to the inhibition of autophagy in myocardial cells at CT8 and CT12. The JTK_CYCLE analysis results revealed that the rhythmic expression of the LC3II protein was lost in the myocardial tissue of actively immunized mice (JTK_CYCLE ADJ, *P*  > 0.05) ( Table 1).

**Table:** **Table 1** Predicting cyclic parameters of myocardial LC3II protein expression with JTK_CYCLE algorithm

Sample type	Protein	Group	JTK_CYCLE *P* value	Circadian JTK_CYCLE
*In vivo*	LC3II	Control	0.04	Yes
*In vivo*	LC3II	β _1_-AA	0.85	No
*In vitro*	LC3II	Control	0.045	Yes
*In vitro*	LC3II	β _1_-AA	8.13e-06	Yes

Then, 100 nM dexamethasone was used to induce the synchronization of H9c2 cardiomyocytes. The flow cytometry results revealed that the proportion of cells in DipG1 (G0/G1 phase) increased from 52.28% to 71.21% after 4 h of treatment with dexamethasone, suggesting that the cells had reached synchronization ( Figure 1C). Further detection of LC3II protein expression in cardiomyocytes revealed that LC3II protein expression in cardiomyocytes in the control group had a significant circadian rhythm (JTK_CYCLE ADJ, *P < *0.05) ( Table 1). β _1_-AA induced a decrease in LC3II protein expression in cardiomyocytes at both CT8 and CT12, with the most significant decrease at CT8. The total LC3 protein expression, expressed as (LC3I + LC3II)/β-actin, matched the trend of LC3II (LC3II/β-actin). These findings indicate that β _1_-AA exerts the most pronounced inhibitory effect on myocardial autophagy at CT8 ( Figure 1D). The JTK_CYCLE analysis indicated that LC3II expression maintained circadian rhythmicity ( *P*  < 0.05) under β _1_-AA treatment. However, significant rhythm disruptions were noted, with distinct phase shifts between the groups: the control group had a peak at CT8, while the β _1_-AA group displayed a 4-hour phase advance, peaking at CT4 ( Table 1 and Figure 1D). These results suggested that β _1_-AA disrupted the rhythmic expression of the myocardial autophagy marker protein LC3.

### β _1_-AA disrupts the rhythmic expression of the PER2 protein in cardiomyocytes, mainly by downregulating PER2 protein expression

**Figure 2:**
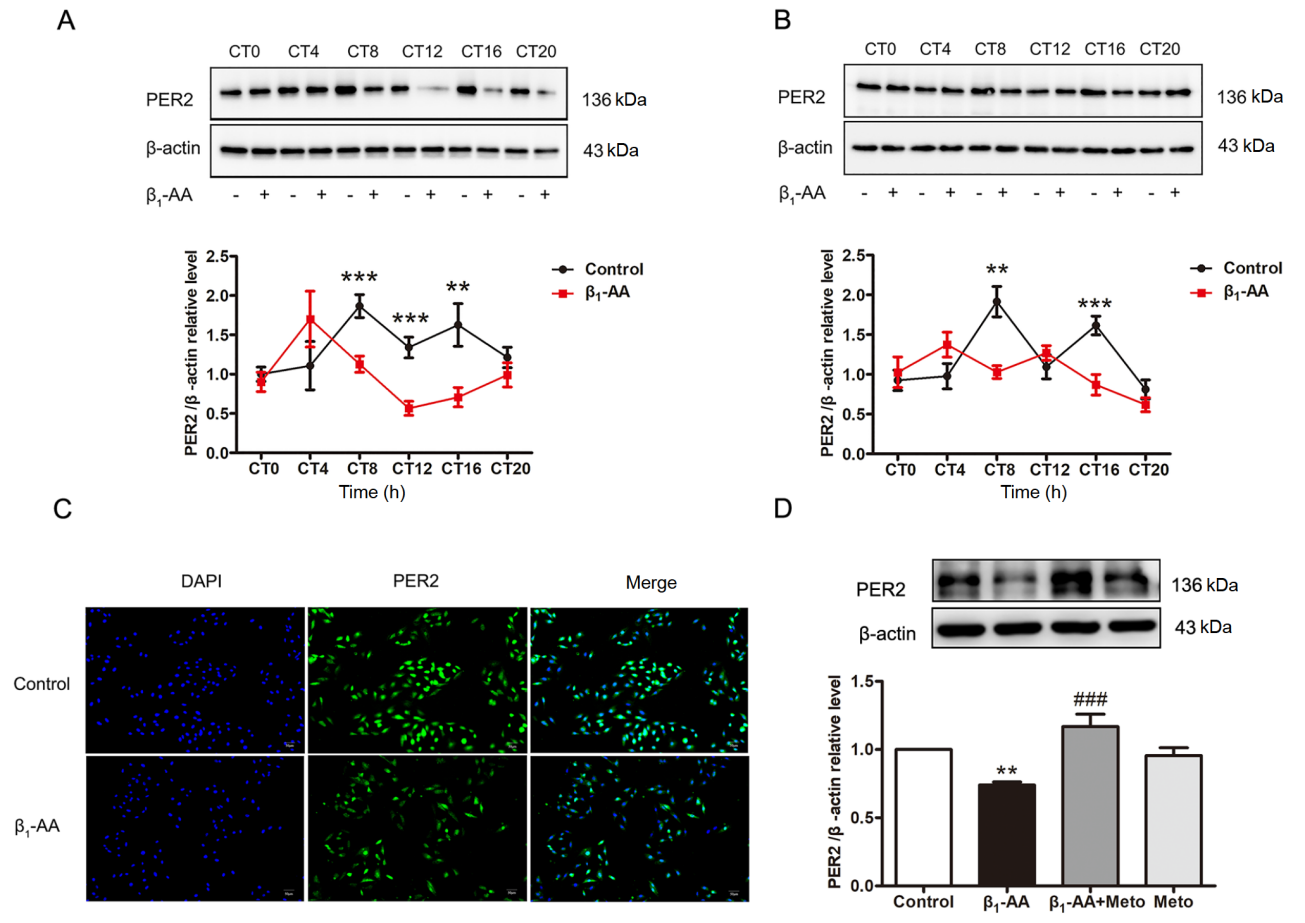
Figure 2 β1-AA inhibits the rhythmic expression of PER2 protein (A) PER2 expression in myocardial tissue of mice in the control group and β1-AA group at different time points (n = 6). (B) PER2 expression in cardiomyocytes of the control group and β1-AA group at different time points (n = 12). (C) The fluorescence intensity of PER2 protein in cardiomyocytes of the control group and β1-AA group (green: FITC-labeled PER2; DAPI: blue marked nucleus). (D) PER2 expression in cardiomyocytes of the control group, β1-AA group, β1-AA+metoprolol group and metoprolol group. **P < 0.01, ***P < 0.001 vs control group; ###P < 0.001 vs β1-AA group.

The results of the western blot and JTK_CYCLE analyses revealed that the protein expression of PER2 in the myocardial tissue of the control group exhibited obvious circadian fluctuations. PER2 protein expression in the myocardial tissue of actively immunized mice was significantly inhibited at CT8, CT12, and CT16 ( Figure 2A). Although *P* value (JTK_CYCLE ADJ) was less than 0.05, indicating a circadian rhythm, the peak value of PER2 expression decreased, and the phase moved forward, suggesting that β _1_-AA could lead to rhythmic changes in PER2 protein expression in myocardial tissue ( Table 2). PER2 protein expression in H9c2 cardiomyocytes in the control group exhibited obvious circadian fluctuations (JTK_CYCLE ADJ, *P*  < 0.01) ( Table 2). β _1_-AA significantly inhibited PER2 protein expression in cardiomyocytes at CT8 and CT16 ( Figure 2B). A *P* value greater than 0.05, according to the JTK_CYCLE ADJ algorithm, suggested that PER2 protein expression in cardiomyocytes did not fluctuate in the circadian rhythm under β _1_-AA treatment ( Table 2). Since both the PER2 protein and the LC3II protein decreased significantly at the CT8 under β _1_-AA treatment, the expression of the PER2 protein in cardiomyocytes at CT8 was further detected by immunofluorescence staining, and the results revealed that β _1_-AA caused a significant reduction in the intensity of green fluorescence (FITC labeling) representing the PER2 protein ( Figure 2C). These results suggested that β _1_-AA could indeed inhibit the expression of the PER2 protein in cardiomyocytes.

**Table:** **Table 2** Predicting cyclic parameters of myocardial PER2 protein expression with JTK_CYCLE algorithm

Sample type	Protein	Group	JTK_CYCLE *P* value	Circadian JTK_CYCLE
*In vivo*	PER2	Control	0.045	Yes
*In vivo*	PER2	β _1_-AA	0.008	Yes
*In vitro*	PER2	Control	0.014	Yes
*In vitro*	PER2	β _1_-AA	0.262	No

Furthermore, we used metoprolol as a specific inhibitor of β _1_-AR, and detected the protein expression levels of PER2 in the control group, β _1_-AA group, β _1_-AA+metoprolol group and metoprolol group. The results indicated that, compared with the control, β _1_-AA significantly decreased PER2 protein expression in cardiomyocytes, whereas metoprolol reversed this decrease. Moreover, metoprolol alone did not affect PER2 expression in cardiomyocytes ( Figure 2D).

### Decreased PER2 protein expression contributes to the downregulated LC3 protein induced by β _1_-AA in cardiomyocytes

**Figure 3:**
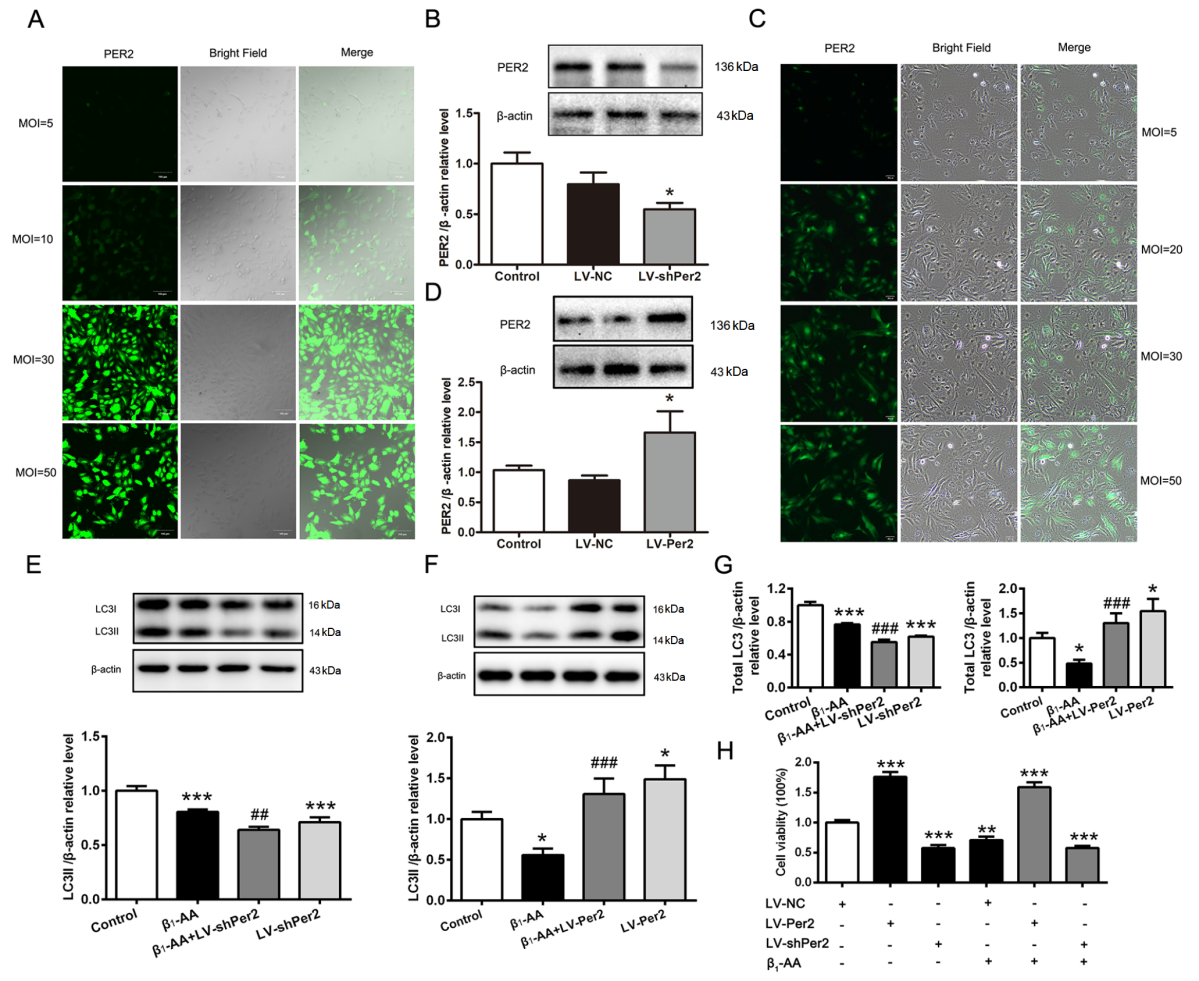
Figure 3 Decreased expression of PER2 protein is involved in the downregulation of LC3II protein expression induced by β1-AA (A) Screening of the best MOI of lentivirus infection for the knockdown of Per2 in cardiomyocytes. (B) Western blot analysis was used to verify the efficiency of Per2 knockdown (n = 4). (C) Screening of the best MOI of lentivirus infection for Per2 overexpression in cardiomyocytes. (D) Western blot analysis was used to verify the efficiency of Per2 overexpressiond (n = 6). (E) Expression of LC3II in different groups when Per2 was knocked down (n = 6). (F) Expression of LC3II in different groups when Per2 was overexpressed (n = 5). (G) Expression of total LC3 in different groups when Per2 was knocked down (n = 6) or overexpressed (n = 5). (H) The survival of cardiomyocytes after knockdown and overexpression of Per2 was analyzed by CCK8 assay. *P < 0.05, **P < 0.01, ***P < 0.001 vs control group; ##P < 0.01, ###P < 0.001 vs β1-AA group.

To confirm the role of the PER2 protein in the reduction in LC3II expression induced by β _1_-AA, we established cell models with *Per2* knockdown or overexpression by lentivirus infection. The optimal multiplicity of infection (MOI) for *Per2* knockdown via lentivirus was determined to be 30 ( Figure 3A). Stable transgenic cardiomyocytes were obtained after treatment with puromycin for 48 h. Western blot analysis revealed a significant reduction in PER2 expression in cardiomyocytes with *Per2* knockdown ( Figure 3B). The optimal MOI for the overexpression of PER2 via lentivirus was 20 ( Figure 3C). Western blot analysis results revealed that PER2 expression was significantly increased in cardiomyocytes with Per2 overexpression ( Figure 3D). Subsequently, the cells were treated with dexamethasone for 4 h for synchronization and then with β _1_-AA for 8 h. Western blot analysis was used to detect the expression of the autophagy marker protein LC3. Compared with the control, β _1_-AA significantly inhibited LC3II protein expression in cardiomyocytes. Additionally, LC3II protein expression was further reduced by β _1_-AA following *Per2* knockdown. LC3II expression in cardiomyocytes was also significantly lower in the *Per2*-knockdown group than in the control group ( Figure 3E). As shown in Figure 3F, *Per2* overexpression significantly reversed this reduction caused by β _1_-AA. Compared with that in the control group, LC3II expression was significantly increased in cardiomyocytes overexpressing Per2 alone. Similar results were obtained for total LC3 expression ( Figure 3G).

Next, the survival rate of cardiomyocytes subjected to *Per2* knockdown or overexpression was analyzed. The results revealed that the survival rate of *Per2*-knockdown cells was significantly decreased, whereas the survival rate of *Per2*-overexpressing cells was significantly increased. Compared with that of β _1_-AA alone, the survival rate of cardiomyocytes was still lower when *Per2*-knockdown cells were treated with β _1_-AA. However, *Per2* overexpression significantly reversed the decreased survival rate of cardiomyocytes induced by β _1_-AA ( Figure 3H), suggesting that the downregulation of PER2 protein expression in cardiomyocytes induced by β _1_-AA promoted cardiomyocyte death by inhibiting autophagy.

### Increased mTORC1 activity in cardiomyocytes is involved in the downregulation of LC3II expression induced by β _1_-AA

**Figure 4:**
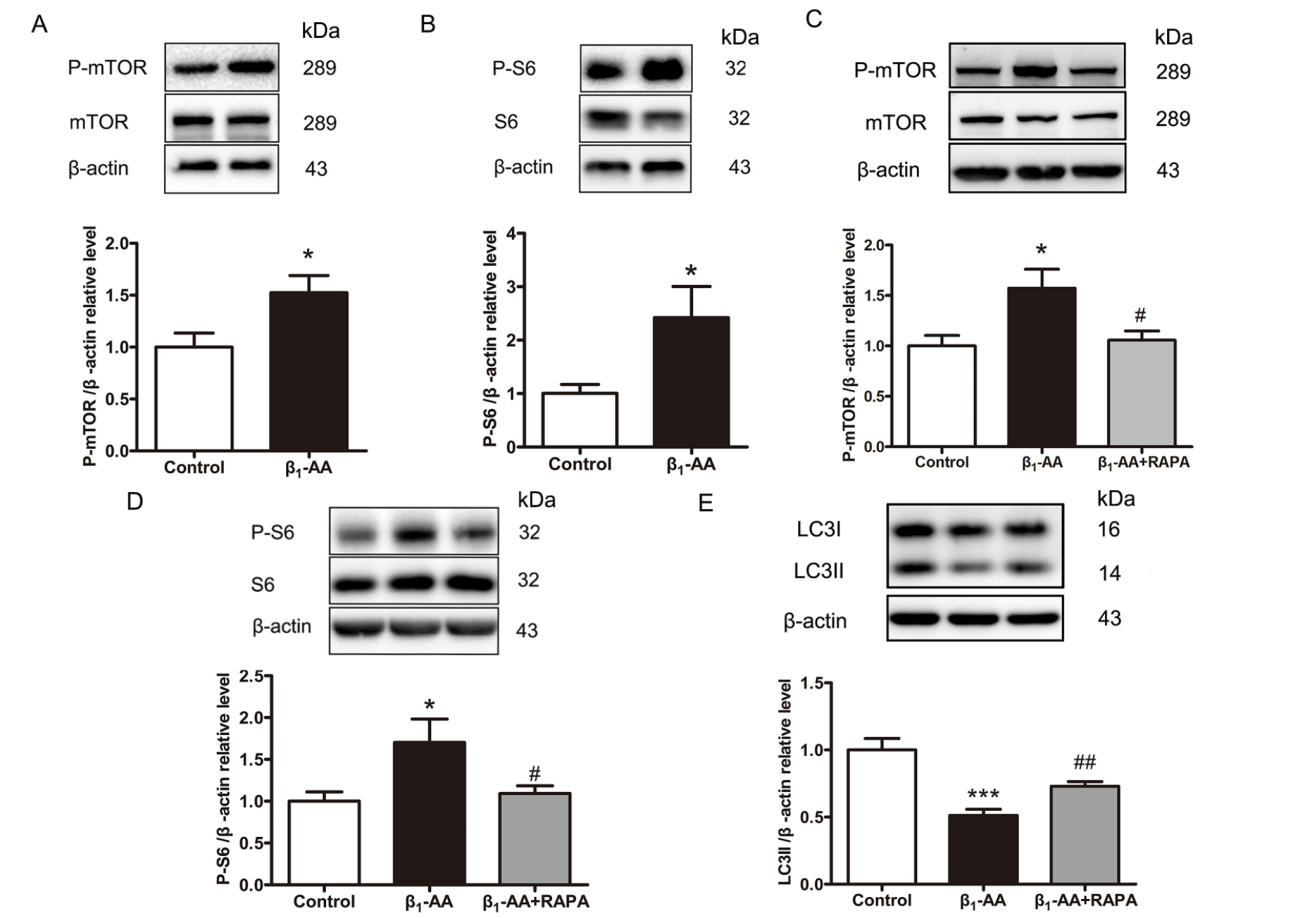
Figure 4 Rapamycin pretreatment improves the β1-AA-induced decline in the expression of autophagy marker protein LC3II in cardiomyocytes (A) Western blot analysis was performed to detect P-mTOR levels at CT8 in cells of control group and β1-AA group (n = 5). (B) Western blot analysis was performed to detect the phosphorylation levels of mTORC1 downstream molecule S6 at CT8 in cells of the control group and β1-AA group (n = 4). (C) Western blot analysis was performed to detect the phosphorylation levels of cardiomyocytes mTOR in the control group, β1-AA group and β1-AA + RAPA group (n = 20). (D) Western blot analysis was performed to detect the phosphorylation levels of cardiomyocytes S6 in the control group, β1-AA group and β1-AA + RAPA group (n = 9). (E) Western blot analysis of LC3II protein expressions in cardiomyocytes of control group, β1-AA group and β1-AA + RAPA group (n = 11). *P < 0.05, ***P < 0.001 vs control group; #P < 0.05, ##P < 0.01 vs β1-AA group.

To explore whether β _1_-AA affects mTORC1 activity, western blot analysis was performed to detect mTORC1 activity in cardiomyocytes at CT8. The phosphorylation of mTOR and its downstream molecule S6 is used to represent the activity of mTORC1. The results revealed that β _1_-AA significantly increased P-mTOR protein level and P-S6 protein level in cardiomyocytes ( Figure 4A,B). These findings suggest that β _1_-AA could induce an increase in mTORC1 activity in cardiomyocytes.

These findings confirmed that mTORC1 activation is involved in the process of the inhibition of autophagy induced by β _1_-AA. We first synchronized the cardiomyocytes with dexamethasone for 4 h, then we added rapamycin for a 2-h pretreatment during synchronization. Finally, we added β _1_-AA following the completion of synchronization. Compared with β _1_-AA treatment alone, rapamycin pretreatment significantly inhibited the protein levels of P-mTOR and P-S6, suggesting that rapamycin could inhibit the mTORC1 activity ( Figure 4C,D). Moreover, pretreatment with rapamycin followed by β _1_-AA intervention significantly reversed the β _1_-AA-induced decrease in LC3II protein expression in cardiomyocytes ( Figure 4E). These results suggest that mTORC1 activation is indeed involved in the inhibition of myocardial autophagy induced by β _1_-AA.

### β _1_-AA promotes mTORC1 activity by inhibiting PER2 expression in cardiomyocytes

**Figure 5:**
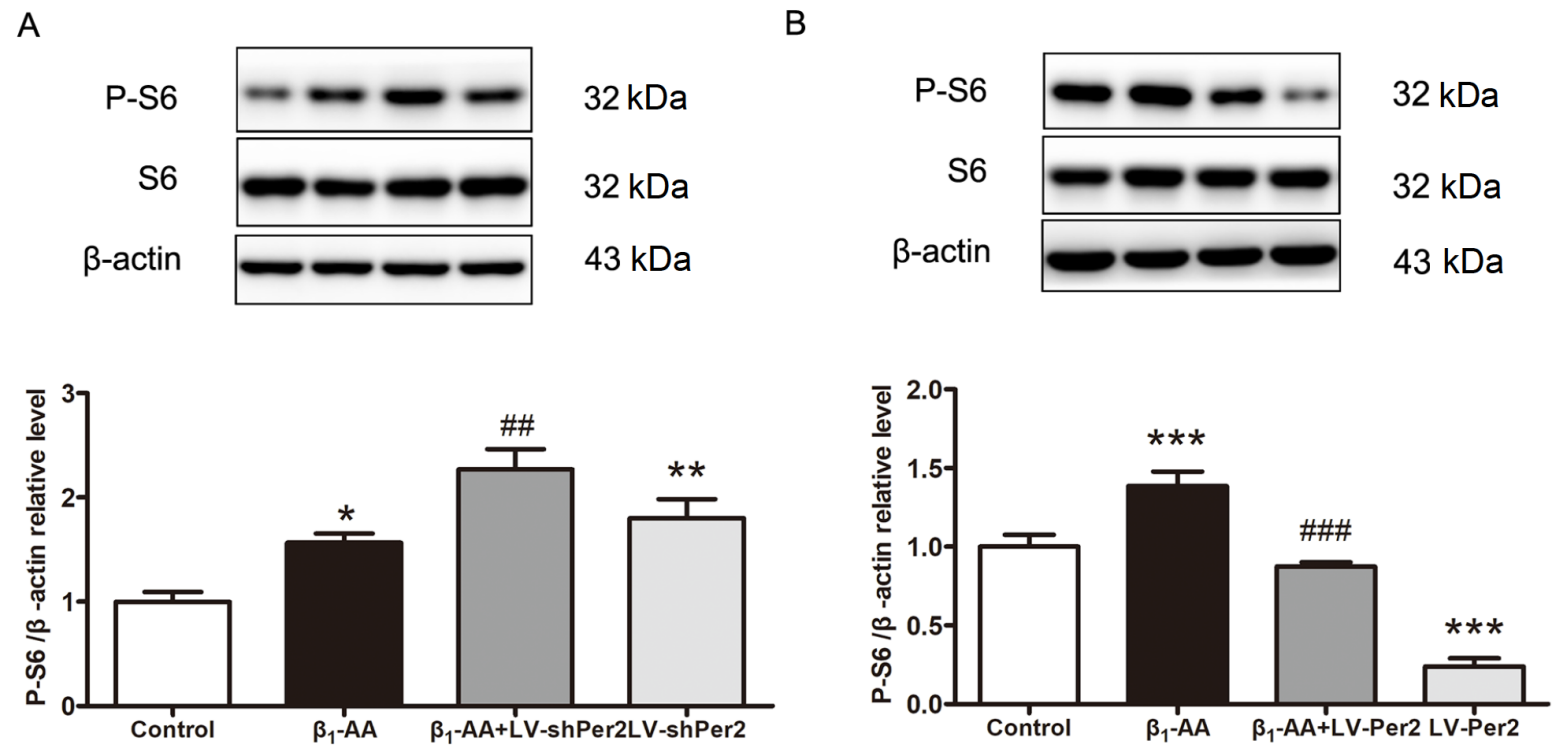
Figure 5 Downregulated PER2 expression is involved in increased mTORC1 activity in cardiomyocytes induced by β1-AA (A) The levels of P-S6 in cardiomyocytes of LV-NC group, LV-NC + β1-AA group, LV-shPer2 + β1-AA group and LV-shPer2 group were detected by western blot analysis (n = 6). (B) Western blot analysis of cardiomyocyte S6 phosphorylation levels in LV-NC group, LV-NC + β1-AA group, LV-Per2 + β1-AA group and LV-Per2 group (n = 6). *P < 0.05, **P < 0.01, ***P < 0.001 vs control group. ##P < 0.01, ###P < 0.001 vs β1-AA group.

To investigate whether the decrease in PER2 protein in cardiomyocytes affects the activity of mTORC1 under β _1_-AA treatment, we used lentiviruses to knockdown or overexpress *Per2* in cardiomyocytes and detected the phosphorylation level of the mTORC1 downstream molecule S6 via western blot analysis. Compared with the β _1_-AA group, *Per2* knockdown in cardiomyocytes further increased the β _1_-AA-induced P-S6 protein level, and *Per2* knockdown alone significantly increased P-S6 protein level in cardiomyocytes compared with that in the control group ( Figure 5A). Overexpression of *Per2* significantly decreased P-S6 protein level in cardiomyocytes compared with the β _1_-AA group and *Per2* overexpression alone decreased P-S6 protein level significantly compared with the control group ( Figure 5B). These findings suggested that β _1_-AA promoted an increase in mTORC1 activity by inhibiting PER2 expression in cardiomyocytes.

## Discussion

Previous studies have shown that autophagy can maintain cell homeostasis and reduce cell death, and the number of autophagic vacuoles in cardiomyocytes fluctuates rhythmically [ 15, 16]. New statistics have shown that most major heart disease outbreaks, such as heart failure, sudden cardiac death, and myocardial infarction [17], occur in the early morning [ 18, 19] when autophagy activity is low throughout the day, suggesting that the occurrence of heart failure may be related to fluctuations in the rhythm of autophagy during the day. Studies have shown that disturbance of the autophagy rhythm can cause the death of cardiomyocytes, which is one of the important reasons for the induction of heart failure [9]. Therefore, it is necessary to identify the factors that disturb the autophagy rhythm of cardiomyocytes during heart failure.

Clinical data have shown that an autoantibody, known as β _1_-AA, that persistently activates the β _1_-AR is present in the serum of 40%–60% of heart failure patients. β _1_-AA can continuously activate β _1_-AR on the surface of cardiomyocytes and induce autophagy inhibition, resulting in cardiac cell death and cardiac insufficiency [ 20, 21]. In this study, we confirmed that β _1_-AA could disturb the autophagy rhythm of cardiomyocytes. First, we established a β _1_-AA-positive mouse model via active immunization with the β _1_-AR-ECII antigen peptide. During this period, the mice were subjected to synchronous treatment of L/D for 2 weeks and then to D/D for another 2 weeks. Specifically, the mice were initially kept under a 12-h light/12-h darkness (LD) cycle for 14 days to establish diurnal entrainment and then kept in continuous darkness (DD) to eliminate light interference and observe endogenous rhythm changes [ 22, 23]. The results revealed that the autophagy rhythm was significantly disrupted in the myocardial tissue of the mice, mainly as the expression of the autophagy marker protein LC3II decreased significantly in at CT8 and CT12. The JTK_CYCLE algorithm was used to analyze the circadian rhythm parameters of protein expression, and the results revealed that LC3II rhythm expression was lost in the myocardial tissue of actively immunized mice. H9c2 cardiomyocytes were subsequently treated with dexamethasone (final concentration 100 nM) for 1, 2, and 4 h. Dexamethasone, a synthetic glucocorticoid, activates the glucocorticoid response element on the clock gene promoter. This regulation influences peripheral rhythms, causing most cells to be arrested in the G0/G1 phase, resulting in cell synchronization [ 24, 25]. Flow cytometry was used to detect the proportions of cells in different stages of the cell cycle. The results revealed that the proportion of cardiomyocytes in the G0/G1 phase reached 71.21% after dexamethasone treatment for 4 h, reflecting a good synchronization effect [ 26– 29]. Furthermore, autophagy was detected in synchronized cells, and studies have shown that changes in LC3II level and total LC3 level can be used as indicators of autophagy [30]. After 4 h of dexamethasone treatment, β _1_-AA significantly decreased LC3II protein expression and total LC3 protein expression in cardiomyocytes at CT8 and CT12. JTK_CYCLE analysis revealed that although LC3II rhythm expression was still present in cardiomyocytes treated with β _1_-AA, the peak value decreased, and the phase advanced. These results suggested that β _1_-AA could indeed disturb the autophagy rhythm of cardiomyocytes. However, how β _1_-AA induces dysrhythmia during autophagy remains unclear.

The biological clock and autophagy are known to be involved in regulating many physiological processes. Increasing evidence shows that the circadian clock can regulate the rhythm of autophagy [ 10, 31]. Among them, the core protein of the biological clock PER2 plays a crucial role in regulating the body’s circadian rhythm. Isoproterenol, a β-adrenergic receptor agonist, can regulate the rhythmic expression of *Per2* [32]. Moreover, overexpression of *Per2* has been shown to improve myocardial infarction by increasing autophagy level [13]. Therefore, is β _1_-AA-induced autophagy rhythm disturbance related to *Per2* regulation? The results of this study revealed that rhythmic PER2 expression in the myocardial tissue of active immune model mice was disrupted, as the peak value decreased and the phase advanced. β _1_-AA intervention in synchronized cardiomyocytes significantly inhibited the rhythmic expression of the PER2 protein in cardiomyocytes, especially at CT8 and CT16. JTK_CYCLE analysis revealed that the expression of the PER2 protein does not exhibit circadian fluctuations. Since both the PER2 protein and the LC3II protein decreased significantly at CT8 under the action of β _1_-AA, the CT8 time point was adopted for subsequent experiments. Then, *Per2*-overexpressing and *Per2*-knockdown lentiviruses were used to infect cardiomyocytes, and the results revealed that at CT8, *Per2* knockdown decreased LC3 protein expression and the cell survival rate, whereas Per2 overexpression significantly reversed the decrease in LC3 expression and the survival rate of cardiomyocytes induced by β _1_-AA. These results indicated that β _1_-AA disturbs the autophagy rhythm by inhibiting the expression of *Per2* and eventually leads to the death of cardiomyocytes. Next, we investigated how Per2 downregulation is involved in the β _1_-AA-induced inhibition of autophagy.

Studies have shown that the PER2 protein in the mouse liver can regulate autophagy by inhibiting mTORC1 activity [33]. The mTORC1 signaling pathway is one of the classical pathways regulating autophagy [34]. The mTOR complex is divided into mTORC1 and mTORC2. mTORC1 is composed of the mTOR, Raptor, Deptor, mLST8 and PRAS40 subunits. It can inhibit the phosphorylation of UNC-51-like autophagy-activating kinase 1 (ULK1) by AMPK, resulting in the failure of LC3II, which is necessary for the formation of autophagosomes and thus inhibits autophagy [34]. An increasing number of studies have shown that abnormal mTORC1 activation is a common phenomenon in many diseases, including heart failure [ 35, 36]. Thus, we speculate that mTORC1 activation may be involved in the disturbance of the myocardial autophagy rhythm induced by β _1_-AA. Western blot analysis revealed that β _1_-AA could increase P-mTOR and P-S6 levels in myocardial tissue and cells at CT8, suggesting that β _1_-AA could activate mTORC1 activity in cardiomyocytes. To further clarify the role of mTORC1 activation in β _1_-AA-mediated inhibition of autophagy at CT8, we used the mTORC1 inhibitor rapamycin to pretreat cardiomyocytes and then performed β _1_-AA intervention. The results showed that rapamycin significantly reversed the decreased expression of the LC3II protein induced by β _1_-AA. β _1_-AA could indeed induce autophagy inhibition by activating mTORC1 at CT8. Furthermore, Per2 downregulation increased β _1_-AA-induced P-S6 level, whereas Per2 overexpression reversed the β _1_-AA-induced P-S6 increase. These results suggested that the downregulation of PER2 expression induced by β _1_-AA could inhibit autophagy by activating mTORC1 in cardiomyocytes.

**Figure 6:**
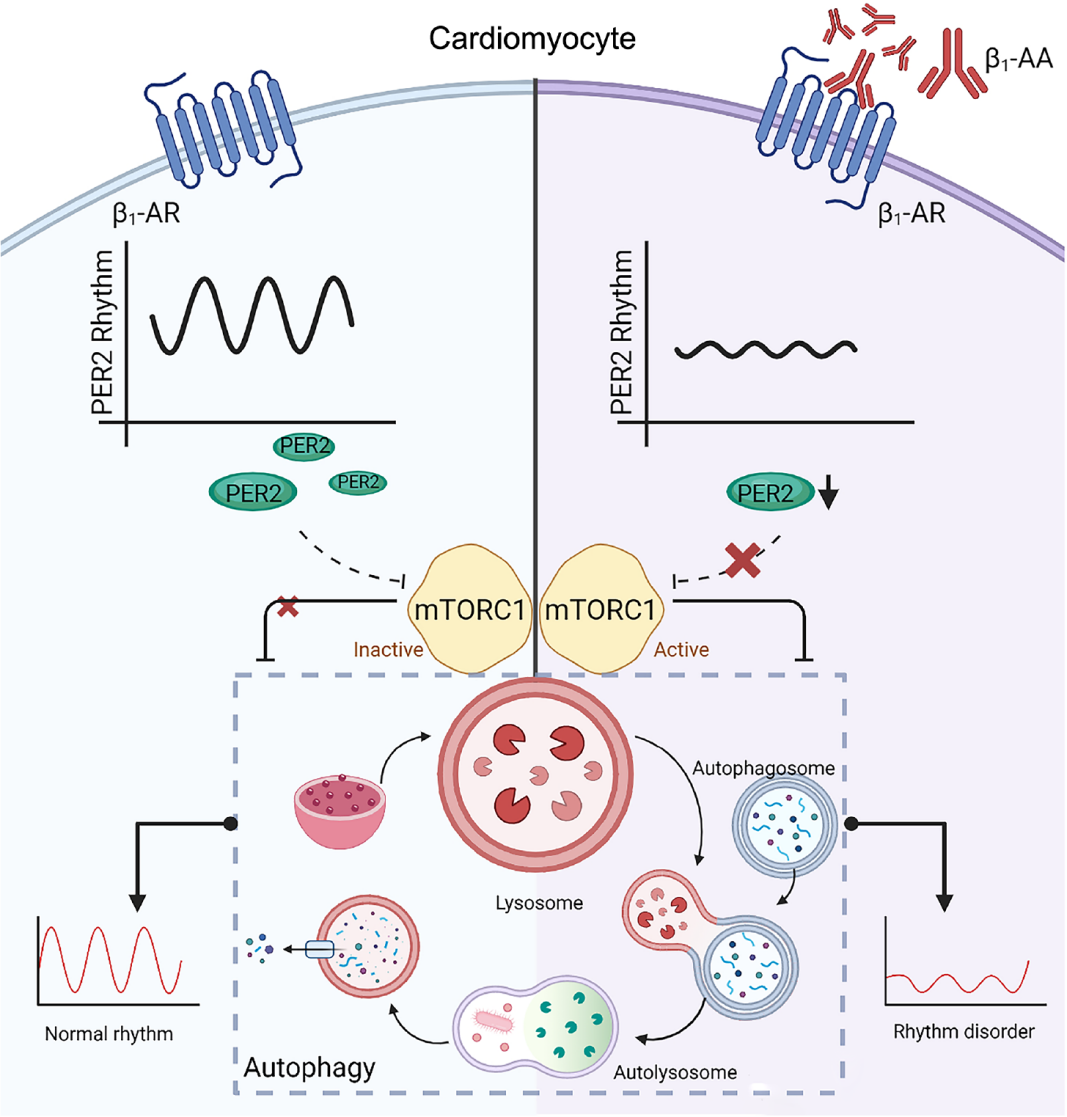
Figure 6 A working model illustrates how β1-AA induces cardiac autophagy rhythm disorders By inhibiting PER2 expression, β1-AA ultimately inhibits the expression of autophagy marker protein LC3 in cardiomyocytes. β1-AA decreases the expression of PER2 in cardiomyocytes. Then, the downregulation of PER2 under the action of β1-AA induces the disruption of autophagy rhythm by promoting mTORC1 activation. This model is created with BioRender.com.

In summary, mTORC1 activation caused by the downregulation of PER2 protein expression in cardiomyocytes is an important mechanism of autophagy inhibition induced by β _1_-AA and thus the disturbance of the autophagy rhythm ( Figure 6). The upregulation of PER2 protein expression may be an important means to ameliorate the disturbance of the rhythm of autophagy induced by β _1_-AA and thus improve cardiac function. This study provides an experimental basis for the precision treatment of cardiovascular diseases from the perspective of biological rhythm.
